# 208 Correlates of sedentary behaviour and physical activity in individuals with Crohn’s disease

**DOI:** 10.1093/eurpub/ckae114.052

**Published:** 2024-09-26

**Authors:** Jason Wilson, Barry Lynch, Nathan Graham, Conor McClean, Mark Tully

**Affiliations:** School of Sport, Ulster University, Derry/Londonderry, United Kingdom; School of Sport, Ulster University, Derry/Londonderry, United Kingdom; School of Sport, Ulster University, Derry/Londonderry, United Kingdom; School of Sport, Ulster University, Belfast, United Kingdom; School of Medicine, Ulster University, Derry/Londonderry, United Kingdom

## Abstract

**Purpose:**

Evidence suggests that being physically active could offer a range of benefits for those with Crohn’s disease. However, there is a need to extend this evidence base to increase certainty in how physical activity may provide benefits in terms of quality of life, mental health and wellbeing. There is also a need to examine the correlates of physical activity in Crohn’s disease, and the role reducing levels of sedentary behaviour might have on the health status in this specific population. This study aimed to explore the correlates of sedentary behaviour and physical activity in individuals with Crohn’s disease.

**Methods:**

Adults with Crohn’s disease from the UK and Ireland completed an online survey. Participants completed questions on: demographic characteristics; physical activity; sedentary behaviour; Crohn’s disease severity; health-related quality of life (QOL); anxiety and depressive symptoms; and mental wellbeing. Multiple linear regression analysis using forward selection based on likelihood ratio statistics explored the correlates of sedentary behaviour and physical activity.

**Results:**

One-hundred and eleven individuals (78% female) completed the survey. For sedentary behaviour, mean time was 9.40±3.20 hours/day and the only correlate was age (β = −0.07, t(107) = −2.65, p = 0.009). For both total (mean MET/minutes=2251.49±2363.78) and vigorous physical activity, the QOL physical domain was the only correlate (β = 29.14, t(107)=2.53, p = 0.013 and β = 23.10, t(107)=3.55, p = <0.001, respectively). For moderate physical activity, sex was the only correlate (β = −510.54, t(106) = −2.68, p = 0.009). For walking, there were no correlates.

**Conclusions:**

Higher levels of daily sedentary behaviour were associated with lower age, potentially due to the type of work younger participants were undertaking (i.e. office-based jobs). Higher levels of total physical activity were associated with higher QOL physical domain scores, which demonstrates the potential role partaking in physical activity might have in improving quality of life in individuals with Crohn’s disease. There is a need for more research using device-based tools (i.e. accelerometry) to more accurately measure sedentary behaviour and physical activity in individuals with Crohn’s disease, as these might be useful lifestyle variables to target for health improvement.

**Support/Funding Source:**

This study received no funding, but was supported through advertising by Crohn’s & Colitis Ireland and IBD Relief.

